# Study protocol: Project 2VIDA! SARS-CoV-2 vaccine intervention delivery for adults in Southern California

**DOI:** 10.3389/fpubh.2024.1291332

**Published:** 2024-03-14

**Authors:** Britt Skaathun, Linda Salgin, Fatima A. Muñoz, Gregory A. Talavera, Davey M. Smith, Jamila K. Stockman, Sophie E. O’Bryan, Daniel Ramirez, Cynthia James-Price, Argentina E. Servin

**Affiliations:** ^1^School of Medicine, Division of Infectious Diseases and Global Public Health, University of California, San Diego, La Jolla, CA, United States; ^2^San Ysidro Health Center, San Diego, CA, United States; ^3^Multicultural Health Foundation, San Diego, CA, United States

**Keywords:** COVID-19, African American, Latino (Hispanic), vaccine hesitancy, community-based participatory research

## Abstract

**Background:**

To date, the United States (US) leads the world in the number of infections and deaths due to the Coronavirus Disease 2019 (COVID-19). Racial and ethnic disparities in COVID-19 morbidity and mortality are staggering. Age-adjusted data show that AA and Latino individuals have had higher rates of death over most of the pandemic and during surges. Project *2VIDA!* is community-based participatory research (CBPR) that was developed to address individual, social, and contextual factors related to access and acceptance of the COVID-19 vaccine among African American and Latino communities in Southern California. This paper describes the study protocol and overarching objectives.

**Methods and design:**

Project *2VIDA!* is a multilevel intervention that builds on the principals of CBPR and is designed to increase uptake of the COVID-19 vaccine among African American and Latino individuals (≥16 years and older) in San Diego County. The intervention was developed with a working group comprised of representatives from community and academia and centers on targeted COVID-19 individual awareness and education, linkage to medical and supportive services, COVID-19 community outreach and health promotion and offering the COVID-19 vaccine through community pop-up clinics.

**Discussion:**

Findings from *2VIDA!* will provide data on the impact, feasibility, and acceptability of the intervention which are all crucial for the adaptation, refinement, and improvement of vaccine outreach interventions for COVID-19 and other vaccine preventable infectious diseases that severely impact African American and Latino communities.

**Clinical trial registration:**

https://clinicaltrials.gov/ct2/show/NCT05022472?term=Project+2VIDA&draw=2&rank=1, NCT05022472.

## Introduction

To date, the United States (US) leads the world in the number of infections and deaths due to the Coronavirus Disease 2019 (COVID-19), with 103 million infections and 1.1 million deaths (1). Racial and ethnic disparities in COVID-19 morbidity and mortality are staggering. Early in the pandemic, there were large racial disparities in COVID-19 cases, however, age-adjusted data show that African American, Latino and American Indians and Alaska Natives individuals have had higher rates of death compared with White individuals over most of the pandemic and during surges. Further, research indicates that pre-existing conditions such as diabetes, hypertension, and obesity increase a patient’s risk for severe COVID-19 disease and mortality (2–4). African American and Latino individuals have a disproportionately high prevalence of such comorbidities that are compounded by social and contextual factors such as lower access to healthcare and higher rates of poverty (5–7). Racial and ethnic minority groups also comprised a disproportionate percentage of workers in essential industries (e.g., “front line” employees such as caregivers in nursing homes, transportation, food service) making it more likely for these communities to acquire and transmit the virus as they had limited opportunities to work remotely (8–10). These occupational hazards were intensified for those in the hospitality industry, as only 55% of those workers have access to paid sick leave (10). Within communities of color, there is often higher housing density, more housing insecurity, increased exposure to air pollution, and a greater number of multigenerational households which makes physical distancing harder, thereby increasing the risk of COVID-19 acquisition and transmission (6, 11). These important medical, social, economic, environmental, and political contexts predate and were exacerbated by the pandemic, contributing to disproportionate infections and deaths among African American and Latino individuals in the United States (12, 13).

Furthermore, public confidence in COVID-19 vaccines has been a complex and evolving issue since the vaccines were first developed and rolled out. Several factors have influenced public perception and confidence in these vaccines, including the unprecedented speed at which COVID-19 vaccines were developed, safety and efficacy concerns, vaccine misinformation, and mistrust in the healthcare system and government, among others (14–16). Early in the COVID-19 pandemic, the vaccination rates among African American and Latino individuals lagged well behind that of White individuals (17). Disparities in the uptake of at least one COVID-19 vaccination dose have narrowed over time. According to the CDC, over 8 in 10 people had received at least one COVID-19 vaccination dose as of February 23, 2023 (18). Despite this progress, a vaccination gap persists, particularly among African American individuals. Approximately half (59%) of African American individuals had received at least one dose compared with 64% of White individuals, and 67% of Latino individuals (19–21). Overall, few people have received the updated bivalent booster vaccine dose. Likewise, African American and Latino individuals are about half as likely as White individuals to have received this booster.

Since the onset of the pandemic, a variety of approaches have been employed to improve COVID-19 vaccination rates among racial/ethnic minorities and vulnerable populations, including provider delivered educational interventions (22); patient education, incentives, reminders (e.g., text messaging), motivational interviewing (23); digital interventions (24); community-based approaches (25, 26) and provision of vaccines in settings serving high risk populations (27). However, these studies have yield mixed findings, noting that some interventions had no notable change in COVID-19 vaccine uptake (23). To effectively address vaccine hesitancy among African American and Latino communities, strategies should be culturally sensitive, community-centered, and built on trust. Engaging with trusted community leaders, healthcare providers, and organizations is essential. Additionally, providing accurate information about vaccine safety and efficacy, addressing concerns, and acknowledging historical injustices are key components of any effort to increase vaccination rates among communities of color. Ultimately, promoting vaccine confidence within the African American and Latino community is a crucial step toward controlling the spread of COVID-19 and advancing health equity in the U.S. Building on the lessons learned from the previously described efforts, in December of 2020, utilizing the principals of community based participatory research (CBPR), we formed an intervention working group comprised of representatives from community and academic organizations to address challenges in COVID-19 vaccination uptake among African American and Latino communities in Southern California by using a community-based participatory research (CBPR) approach. Specifically, we have developed Project *2VIDA!* (SARS-CoV-2 Vaccine Intervention Delivery for Adults in Southern California), a multilevel intervention, to combat COVID-19 health misinformation and address individual, social, and contextual factors related to access, acceptance, uptake, and series completion of the COVID-19 vaccine among African American and Latino individuals (≥16 years old) in Southern California. The overall aim of the study is to assess the impact of an intervention known as Project *2VIDA!* focused on addressing vaccine hesitancy and increasing access, acceptance, uptake, and series completion of the COVID-19 vaccine per CDC recommendations among African American and Latino individuals (≥16 years and older) across six communities in Southern California. More specifically, the study will: (1) Assess intervention effects on COVID-19 vaccination rates and series completion among African American and Latino individuals (≥16 years old) living in San Diego County; (2) Determine feasibility and acceptability of the *2VIDA!* intervention among African American and Latino individuals (≥16 years old) living in San Diego County; (3) Examine individual and structural barriers to COVID-19 vaccination among African American and Latino and AA individuals (≥16 years old) living in San Diego County; and (4) Identify the main sources of COVID-19 information that African American and Latino individuals (≥16 years old) trust and are utilizing, to inform efforts to combat health misinformation related to COVID-19 as well as future public health emergencies.

## Methods

### Study design

This is a multicentric cluster randomized controlled trial with a control group and an intervention group, with participant blinding. This protocol has been written according to the recommendations of the SPIRIT 2013 statement, a guideline that provides evidence-based recommendations for a clinical trial protocol, including recommendations for intervention trials. Additionally, the design of this clinical trial follows the requirements of the CONSORT statement.

#### Participants, recruitment, and study settings

**Figure 1:**
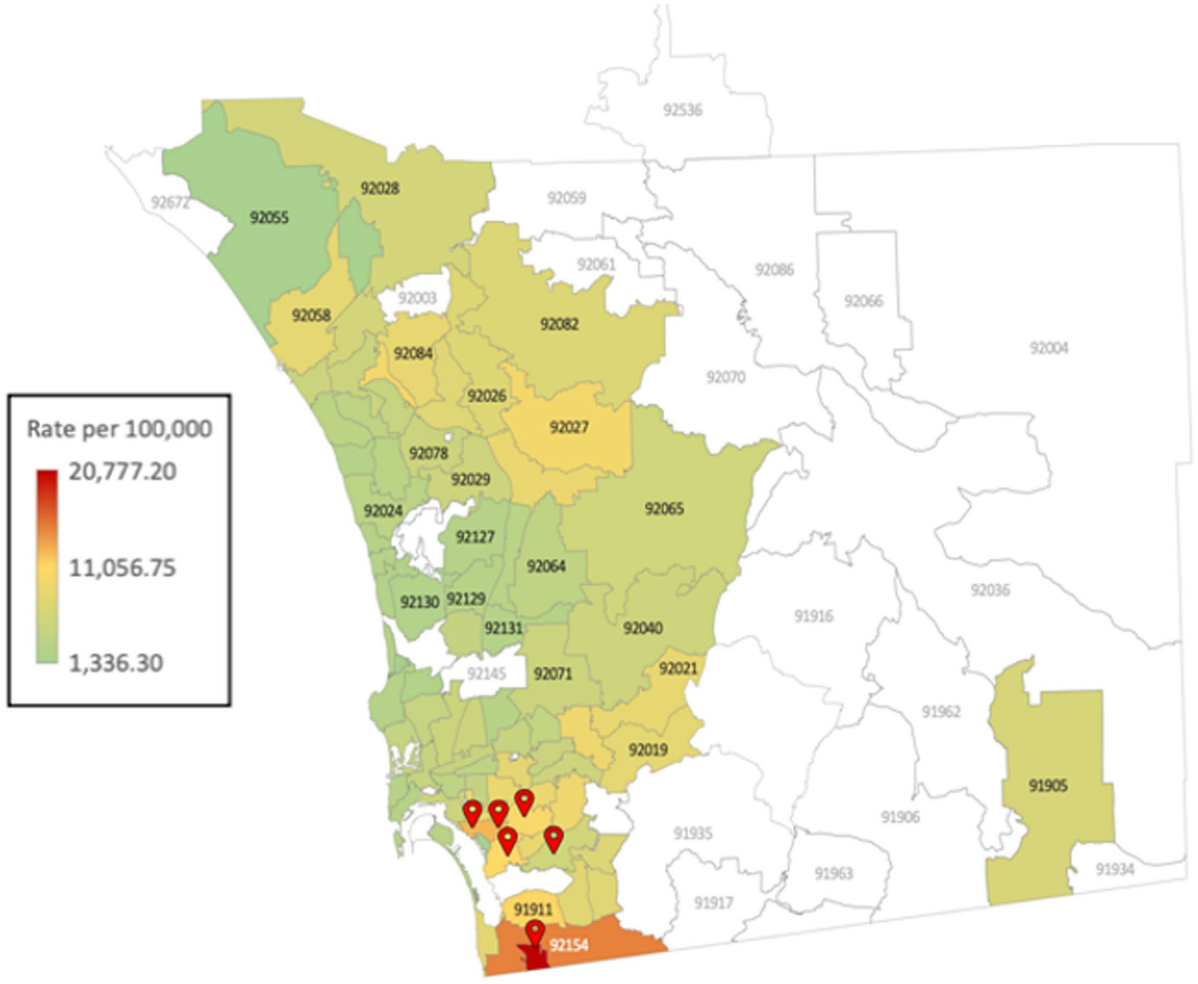
Map of San Diego County with the daily Coronavirus Disease (COVID-19) summary of cases by zip code of residence (data from 01/28/2021) and Project 2VlDA sites.

With a population of 39.2 million people (28), California is home to a many distinct communities. Though all of these communities are the supposed equal beneficiaries of the state public health system, appreciable inequity is evident. In mid-2020, one of the most salient factors dividing local communities, unfortunately, remains racial composition. Southern California is one of the most ethnically diverse areas that was hit the hardest during the pandemic (29, 30). However, in San Diego County, the impact of the virus varied dramatically depending on ZIP code. For example, population and case-rate data indicate that individuals that lived in zip codes associated to more affluent neighborhoods such as Carmel Valley (located 30 miles north from San Ysidro), were nearly 60 times less likely to live next to someone who tested positive for COVID. Compared to individuals in San Ysidro, were one in every 10 individuals tested positive (31). These ZIP codes were concentrated in Southeast San Diego and home to predominantly racial and ethnic minorities who are uninsured and live below the federal poverty line (32). Participants were recruited from the zip codes that reported the highest number of cases in San Diego County, that include the communities of National City, Logan Heights, Lincoln Park, Valencia Park, Chula Vista, and San Ysidro (Figure 1).

Participants from both intervention (*n* = 500) and control sites (*n* = 500) were recruited using multipronged recruitment strategies including the use of trusted community partners, social media platforms, and flyer distribution within the selected health centers and the selected communities in highly trafficked and readily visible areas where eligible participants frequent (e.g., grocery stores, local community-based organizations [CBOs], faith-based organizations, parks, food banks, gyms, local restaurants). Flyers include study and contact information for study staff. Likewise, project staff publicize the community pop-up clinics by posting flyers that note locations, dates, and times of vaccine distribution and through presentations about the project at community meetings. Project staffing, training, and support, including the selection of nurses and outreach staff (e.g., peer-health educators and research assistants) with complementary research skills and personal knowledge of the community were key to the success of the project. Bilingual outreach workers trained in research ethics and protocols, and familiar with the target population, approached individuals who appear to be eligible at the health center, and community pop-up clinics in the participating sites.

Research assistants approached potential eligible participants from the waiting rooms of the health centers and at the community pop-up clinics and assessed whether or not they met the inclusion criteria described below; they explained the purpose of the study including its risk and benefits. They were also reminded that participation in the study was voluntary and if they declined to participate, they would not lose access to care or any other service they were currently receiving or eligible to receive. Research assistants recorded data regarding eligibility and reasons for non-participation where applicable, however, no personal identifying information was collected. These data were recorded in the form of a Client Recruitment Log. Those agreeing to participate were escorted to a private room within the participating health center or at the community pop-up clinic and provided verbal consent and completed the survey via a tablet (self-administered). Extensive efforts were made to have a study sample that represented the racial and ethnic composition of San Diego County, and at least 40% women. Participants received a $20 VISA gift card for completing the baseline survey and additional $20 VISA gift card for completing the follow-up survey.

#### Inclusion criteria

(i) age 16 years or older, (ii) identify as Latinx and/or AA, (iii) biologically male or female, (iv) be a resident of one of the six communities selected for this study (National City, Lincoln Park, Logan Heights, Valencia Park, Chula Vista or San Ysidro), (v) literate in English or Spanish, (vi) no known history of severe allergic reactions to any components of the vaccine, (vii) no history of immune disease, (viii) not currently pregnant, (ix) no plans to move from the area in the following 30 days, (x) able to provide voluntary informed consent, and (xi) able to provide complete contact information for themselves (for follow-up survey, 2^nd^ vaccine and booster shot).

#### Exclusion criteria

(i) under 16 years old, (ii) pregnant women, (iii) adults unable to consent. Although pregnancy is not a contraindication for COVID-19 vaccination, working group members decided that vaccinating pregnant women, especially at the beginning of the pandemic, would not be well received in the target communities.

#### Randomization of sites

Prior to intervention piloting, the six participating communities were randomized to either the intervention (e.g., pop-up community clinic) or control condition (e.g., health center) using a computer-generated random sequence. Community-level randomization was selected to minimize between-arm contamination of intervention and control conditions. Additionally, these communities were at least 10–20 miles apart from each other to further minimize contamination. Sites matched to the control condition were briefly trained in process evaluation and quality assurance procedures. All sites will participate in process and outcome evaluation protocols. Data collection began June 16, 2021 and is expected to be completed in Fall 2023.

#### Description of the intervention

**Figure 2:**
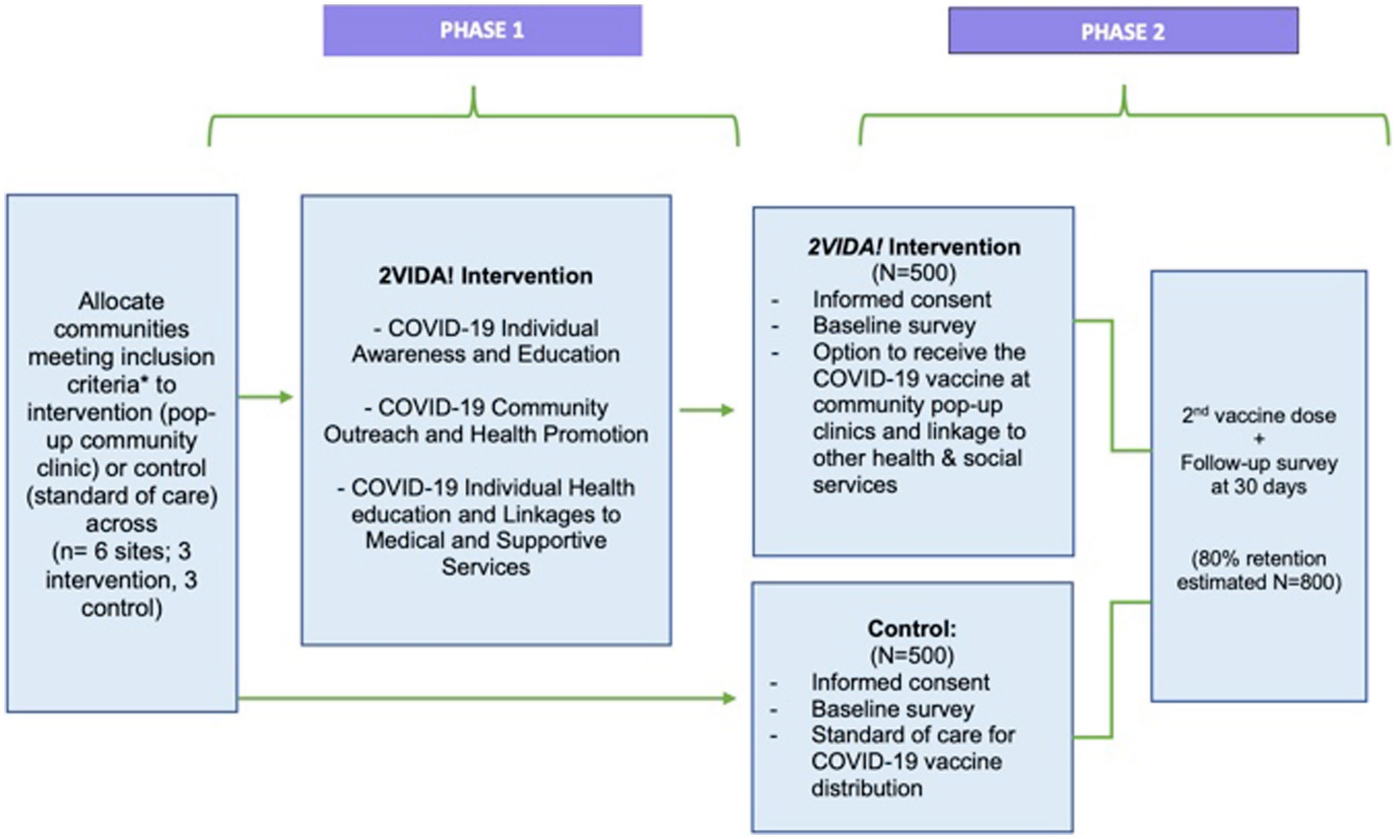
Study design, including the timing of assessments and targeted enrollment. Participants were selected from the communities with the highest number of COVID-19 cases that are also predominantly African American and Latino in San Diego County.

**Figure 3:**
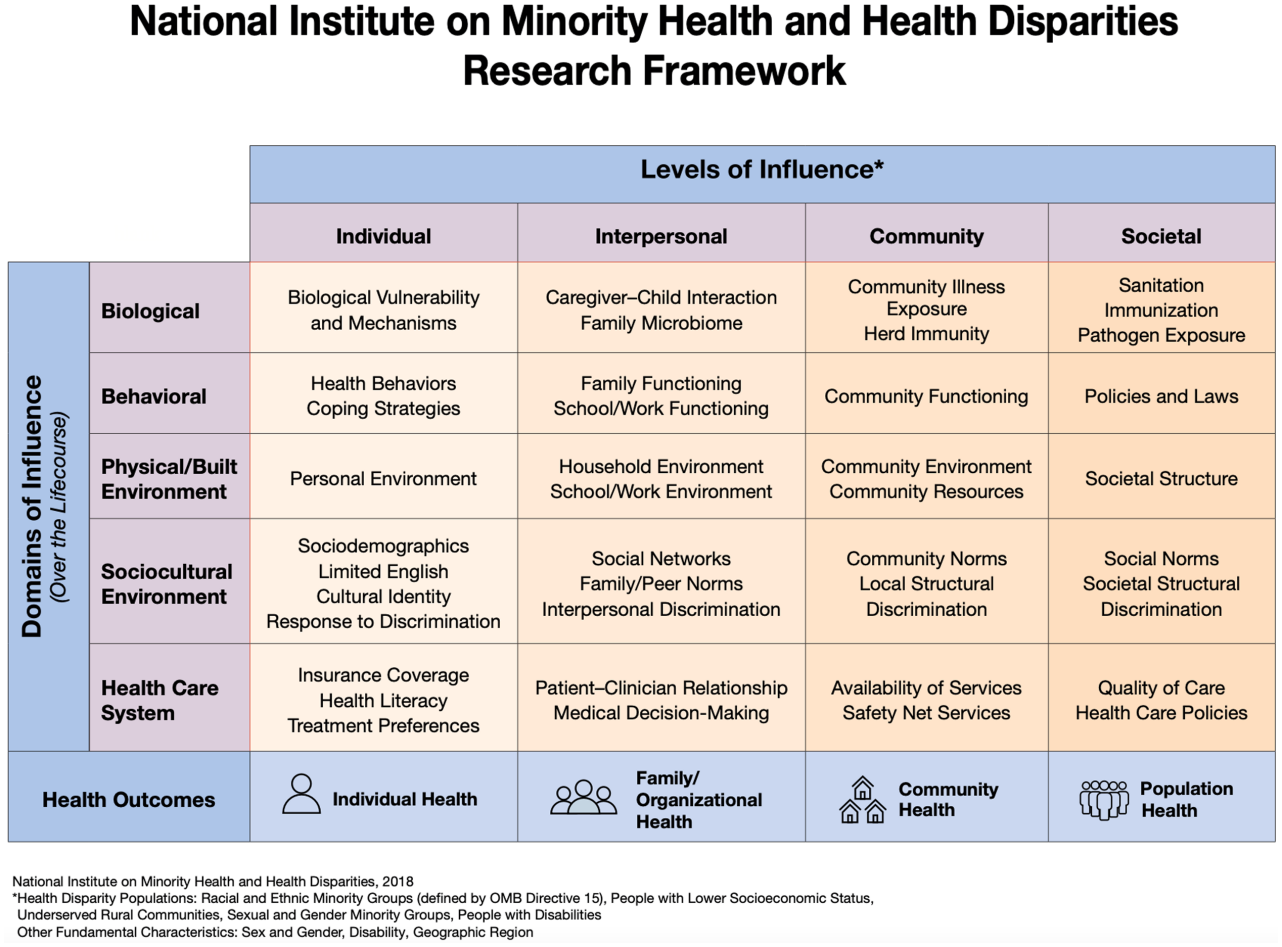
Project *2VlDA!* is a multilevel intervention informed by the National Institute on Minority Health and Health Disparities research framework (33) and the principles of community-based participatory research.

*2VIDA!* is a multilevel intervention informed by the National Institute on Minority Health and Health Disparities (NIMHD) research framework (33) and the principles of CBPR (34, 35). 2*VIDA!* centers on COVID-19 individual awareness and education, linkage to medical and supportive services, COVID-19 community outreach and health promotion and offering the COVID-19 vaccine through community pop-up clinics targeting African American and Latino individuals across six communities in San Diego County (See Figure 2 Study Design). *2VIDA!* is grounded in the NIMHD framework (Figure 3) as it seeks to understand and address health disparities from a multilevel approach by examining individual, social, and contextual factors related to access to, and acceptance of, the COVID-19 vaccine, as well as CBPR. CBPR offers an opportunity to amend health disparities in communities of color. It requires an equitable involvement of researchers and the members of a community that are affected in all aspects of a research process, aiming to improve health, generate knowledge and effect social change. Utilizing the principles of CBPR, we aim to reduce such disparities in health literacy and access to COVID-19 vaccine by addressing the specific challenges of the African American and Latino communities in South San Diego with practical, sustainable, culturally appropriate solutions that utilize the community’s strengths, and test the effectiveness of the intervention by utilizing rigorous research methods. We expect *2VIDA!* to improve health literacy, feasibility, acceptance, and uptake of COVID-19 vaccine among African American and Latino individuals (≥16 years old) in the target communities. The *2VIDA!* intervention has two phases.

#### Phase 1

The phase 1 of the *2VIDA!* intervention has three components outlined below.

##### COVID-19 individual awareness and education

*2VIDA!* formed a CBPR working group that developed culturally competent COVID-19 educational and outreach materials (available in print and digitally) in English and Spanish that were written at an 8th grade reading level (the average reading level of adults in the United States). Peer-health educators distributed to community members during their visits to the participating sites, door-to-door, local supermarkets, and CBOs in the selected communities. These materials have general information on COVID-19 as well as educational information and resources regarding COVID-19 prevention, symptoms, testing, contact tracing, COVID-19 vaccine (how it works, technology used, administration, importance of vaccine series completion and booster), safety concerns, benefits, risks, dispelling common misconceptions and misinformation, and potential side effects, and other topics identified based on community needs. This information is updated on a weekly basis to ensure the most accurate and recent information is provided. Approximately 50, 000 printed educational and outreach materials were distributed across the intervention sites.

##### COVID-19 community outreach and health promotion

Peer-health educators worked with local CBOs to facilitate a combination of targeted live radio broadcast sessions, town hall meetings, power-point presentations in the community and via Zoom, pre-recorded webinars, social media posts, and other outreach activities in English and Spanish in different community settings. These activities engaged community members with information on COVID-19 related topics as the pandemic evolved, as well as other identified needs such as what to do if a family member is infected and where they can get the COVID-19 vaccine. The goal is to reach 10,000 viewers (per session) in the across the social media platforms in the selected intervention communities.

##### COVID-19 individual health education and linkages to medical and supportive services

A COVID-19 Resource Center has been established to serve the intervention sites providing individual COVID-19 related health education and linkages to medical and supportive services for participants and community members in need of additional education and support regarding COVID-19 disease and COVID-19 vaccine.

#### Phase 2

During Phase 2, the COVID-19 vaccine and other health and social are offered through community pop-up clinics that are set-up in the targeted communities as part of our efforts to address some of the barriers Latino and African American communities have to accessing the COVID-19 vaccine. The community pop-up clinics take place in various open spaces such as public parks, parking lots, grocery stores, community recreation centers, swap meets, faith-based organizations, and food banks in these communities.

### Control group (standard of care)

Participants in the control group receive the standard of care for COVID-19 vaccine distribution. As previously mentioned, in order to minimize the occurrence of contamination, randomization occurred at the community level and the intervention sites are located at least 10–1 5 miles or more apart from each other. Data collection surveys to assess individual, social, and contextual factors related to access, acceptance, and uptake of the COVID-19 vaccine be administered at baseline and follow-up.

### Data collection

The baseline survey lasts approximately 20 min and is self-administered via tablet in either English and Spanish. The baseline survey assesses individual, social, and contextual factors related to access, acceptance, and uptake of the COVID-19 vaccine including: (a) sociodemographic characteristics; (b) characteristics associated with social marginalization (e.g., homelessness, food insecurity); (c) access to and utilization of health care; (d) vaccination history, interest, hesitancy and uptake; (e) perceptions of the COVID-19 vaccine (e.g., fears, myths, etc.); (f) general health; (h) trust in government, research, and social agencies; and (i) satisfaction with *2VIDA!* Following participation in the intervention and completion of baseline survey, participants are eligible to receive the COVID-19 vaccine if they are interested and provided informed consent. Participants are also asked to complete a follow-up survey when they receive the second dose or booster. The follow-up survey takes approximately 8 min to complete and assessed changes in (a) access to and utilization of health care; (b) vaccination history, interest, hesitancy and uptake; (c) perceptions of the COVID-19 vaccine (e.g., fears, myths, etc.); (d) trust in government, research, and social agencies; and (e) satisfaction with *2VIDA!* The research staff are always available in case questions arise or help is needed with any aspect of the survey.

### Outcome measures

#### Primary outcome measurement

##### Vaccination hesitancy and distrust

Based on the SAGE Working Group on Vaccine Hesitancy, vaccine hesitancy is defined as “delay in acceptance or refusal of vaccination despite availability of vaccination services” (36).

##### Vaccination interest, uptake, and series completion

Interest in vaccination which is assessed in the survey through the question, “Are you interested in taking a vaccine against COVID-19?”; uptake of COVID-19 which will be recorded as “yes” if they receive the COVID-19 vaccine that day; vaccine series completion will be assessed based on them receiving having completed the series per recommendations by the CDC’s Advisory Committee on Immunization Practices (37) and confirmed through the California Immunization Registry (CAIR).

##### Receipt and acceptability of 2VIDA! intervention

Receipt and acceptability of the intervention was assessed utilizing the Treatment Acceptability and Preferences (TAP) measure tool (38) that contains 4 items scored on a 5-point Likert scale.

### Secondary outcomes measurement

Differences between both groups with respect to the following items:

#### Sociodemographic outcomes

Age (assessed as a continuous measure); sex assigned at birth (male, female, intersex); gender identity (agender, generqueer or genderfluid, man, non-binary, woman); race/ethnicity (American Indian or Alaska Native, Black or African American, Hispanic or Latino, Native Hawaiian or other Pacific Islander, White, Multiracial/more than one race; other); education (never attended school, elementary, some high school, high school graduate, some college or technical school, graduated from college or technical school, postgraduate school); country of origin (this was an open ended question); marital status (single, married, living with a partner, divorced, widow), employment status (employed full time, employed part-time, unemployed, retired, student/currently in school), household income for the past 12 months (less than $5,000, $5,000–$19,999, $20,000 - $49,000, $50,000–$99,999, $100,000–$149,999, More than $150,000), living situation (own, rent, live with friends, live with family, no permanent residence, live at a shelter/currently homeless), household composition (assessed by the question: how many people are currently living in your household. By “living” in your household we mean that they spend more than 2 nights a week in your house).

#### Characteristics associated with social marginalization

Measured by 5 items: Recent and lifetime homelessness, substance abuse, engagement in commercial sex work, food insecurity (adapted from the (39)), and intimate-partner violence (IPV).

#### Medical history

History of having COVID-19 (yes/no) and/or medical conditions (yes/no) that could exacerbate COVID-19 infection including type 1 and type 2 diabetes mellitus, hypertension, heart conditions (e.g., coronary artery disease), obesity (e.g., body mass index of 30 kg/m2 or higher but <40 km/m2), mental health (depression, anxiety, post-traumatic stress disorder), asthma, chronic obstructive pulmonary disease (COPD), and smoking.

#### Access to and utilization of health care

Assessed by 5 items: Insurance status, type of insurance, regularity, and location of access to health care, receipt of services from government or community agencies (adapted from the National Health Interview Survey) (37).

#### Mistrust of COVID-19 vaccine effectiveness

This was assessed by the question “I believe a vaccine can help control the spread of COVID-19,” modeled from the WHO survey (40). This survey item was measured ordinally as a range from “Strongly Agree” to “Strongly Disagree.”

#### Perception of COVID-19 media hype

Using a survey item adapted from the WHO survey (40) participants were asked to rate their perception of COVID-19 being media hyped. Participants’ perceived level of media hype surrounding COVID-19 was measured ordinally along a 7-point rating scale (Strongly Media Hyped to No Media Hype).

#### Frequency of use and level of trust in COVID-19 sources of information

The variables *Level of Trust* and *Frequency of Use* assessed participants’ engagement with several media sources (i.e., Television, Newspapers, Health Workers, Social Media, Radio, Health Departments, the CDC, Celebrities/Influencers, the WHO, COVID-19 Hotlines, National COVID-19 Websites) in relation to COVID-19 information. *Level of Trust* was measured ordinally for each media source as a range from “Very Little Trust” to “A Great Deal of Trust.” *Frequency of Use* was also measured ordinally for each media source from “Never Use” to “Use Very Often” (40).

#### Ease of finding, judging, understanding, and following COVID-19 recommendation

Participants were asked to rate the difficulty level of understanding, judging and following COVID-19 recommendations such as when to stay home, if they needed to get the booster, what symptoms they should look for, etc. These variables were measured ordinally from “Very Difficult” to “Very Easy.”

#### Attitude toward COVID-19 vaccine policies

Participants were asked their attitudes toward public vaccine policies were assessed as categorical variables. Participants were asked to indicate their sentiment toward receiving the vaccine if everyone else was already vaccinated, attitudes toward the national vaccine schedule, and whether they agreed all should follow the recommended guidelines by the government and organizations such as the WHO and the FDA.

#### Withdrawal

Participants may withdraw from the study at any time and by any given reasons. Reasons for withdrawal will be recorded for further study. The participants who withdraw from the study will not be replaced.

#### Adverse events

Any adverse events are currently not foreseen, due to the study and intervention’s nature will be reported accordingly to the IRB and the vaccine adverse event reporting system (VAERS).

### Data analysis

As a feasibility study, the analyses will primarily involve simple univariate and bivariate analyses, with additional logistic and linear regression analyses to explore time x treatment effects from baseline to follow-up. Adjusted regression models will be created to assess whether intervention effects on specified outcomes are maintained subsequent to controlling for potential confounders selected based on baseline treatment group differences in demographics and reported risk factors (e.g., pre-existing conditions, mistrust in government or healthcare system). Findings will receive extensive investigative team and community review to assist with interpretation of findings. The primary outcome of the study is vaccination rate, which is dichotomous. Comparison between the intervention and control arms will be compared using a Fisher’s exact test for proportions. Differences in the rates between the two groups, along with the OR and their 95% CI will be reported. Missing data considerations: Data will be routinely monitored by the study team to ensure completeness. Entered data will be reviewed routinely and inspected for errors, and omissions. A CONSORT diagram will be produced at the end of the study that will show the participant flow in the study, including numbers screened, enrolled, withdrawals, and completers. We expect that the amount of missing data for the primary assessment will be minimal (<15%). Substantial efforts will be made to ensure complete follow-up. Rates of missing data and loss to follow-up will be reported. Missing strategies, such as sensitivity analyses, missing data imputation or propensity weighting, will be considered based on the degree of missingness in the data. Under these various missing data strategies, the statistical analysis will be run and compared for consistency.

### Anticipated results

Results on Project *2VIDA!* feasibility, acceptability, and efficacy to improve COVID-19 vaccination rates and address vaccine hesitancy among African American and Latino individuals (≥16 years old) in San Diego, California will be shared with the scientific community and our community leaders and partners that work with predominately African American and Latino individuals in this region. We anticipate that findings from this study will provide insight on factors that have driven vaccine hesitancy and impacted perceptions of COVID-19, including identifying main trusted sources of COVID-19 information and understanding the individual and structural barriers to accessing the COVID-19 vaccine among African American and Latino communities in Southern California. Furthermore, this data will aid in designing future interventions preventing the spread of misinformation about COVID-19 and how to best communicate and engage with our community to prevent health misinformation. Overall, findings will present key details to assist in preventing the disproportionate and ongoing prevalence of COVID-19 infection as we transition to this endemic phase, as well as prevent long term chronic health implications among African American and Latino communities.

Additionally, a key strength of this intervention is the robust support and involvement of representatives from community and academic organizations. These community-based collaborations allow for sustainability and can be replicated in other settings, ensuring generalizability. Likewise, this collaboration offers expertise in CBPR, health disparities research, and provides systems for disseminating results. All partners will participate in interpretation of findings and dissemination planning, including use of national newsletters and list-servs, participation in conferences and trainings, and creation of presentations and reports for communication to multiple classes of stakeholders. All partners have demonstrated a strong commitment to the proposed study from its inception and will work collaboratively to ensure wide dissemination of the findings of this community practice-based demonstration. Further, publications will be open-access and available after publication under the NIH Public Access Policy in the digital archive PubMed Central. The information derived from *Project 2VIDA!* is expected to offer valuable perspectives for enhancing strategies aimed at addressing COVID-19 vaccine hesitancy and increasing COVID-19 vaccine uptake among our communities of color and the use of CBPR. Lastly, findings can also be applicable to aid in national public health vaccination initiatives to prevent future outbreaks or for other vaccine preventable diseases that significantly impact African American and Latino communities.

## Discussion

The COVID-19 pandemic highlighted racial and ethnic disparities in both infection rates and vaccination coverage, mirroring longstanding health inequities in the United States (2, 41). Tackling this issue amid the urgency of a public health emergency, such as COVID-19, posed numerous challenges, requiring rapid efforts to address decades of unequal healthcare access and the resulting distrust among vulnerable populations. Our strategy was grounded in the principles of CBPR and involved the establishment of a working intervention group comprised of representatives from community, academia, and public health organizations and together we developed *Project 2VIDA!* The response to the project was overwhelmingly positive within the local communities (intervention sites), and community members expressed eagerness to participate in the study (e.g., attend a community engagement forum, a meet the expert Q&A session, receive information about the vaccine in their preferred language) or to be a site for a community pop-up clinic. Previous research has documented the crucial role that community leaders and community-based organizations play in both public health campaigns and vaccination initiatives, contributing significantly to fostering high vaccine uptake and confidence, particularly among racial/ethnic minorities and vulnerable populations (42–44). Likewise, our multilevel intervention integrated COVID-19 educational and outreach materials (printed and digital), utilized different communication channels, community engagement efforts, health promotion, and healthcare provider involvement to combat COVID-19 health misinformation and address individual, social, and contextual factors related to access, acceptance, uptake, and series completion of the COVID-19 vaccine among African American and Latino individuals (≥16 years old) in Southern California. This multifaceted approach has been found effective in enhancing the overall impact on vaccine intervention delivery for adults for other vaccine preventable diseases such as influenza (45, 46).

Although the aim of this project was to target undeserved African American and Latino communities, project staff distributed the vaccine to all interested and eligible persons at the community pop-up clinics, irrespective of individual characteristics. Because the targeted neighborhoods were low-income and many lacked access to healthcare, widespread distribution was warranted.

Our study has several limitations. Data collection efforts began in June 2021, during the period of general eligibility and experiences may have differed compared to when the vaccine first became available to only certain groups. However, this can also be seen as a strength, as the findings are applicable to the present vaccine scenario, where the supply exceeds demand, and everyone is eligible for the vaccine and/or booster. It is also important to acknowledge this study only sampled African American and Latino individuals in San Diego (Southern California), and therefore are not generalizable to other settings, however, the fundamental pillars of the intervention can be adapted to other local contexts.

## Conclusion

The COVID-19 pandemic has been an unprecedented global crisis that has deeply impacted the healthcare sector and revealed several important lessons. Although the WHO ended the public health emergency on May 11, 2023, at the writing of this publication, there has been a significant increase in COVID-19 cases and hospitalization due to the JN.1 variant in the United States and globally (47, 48). With the rise of the JN.1 variant and as we enter winter season when respiratory viruses are known to have a high incidence of infection (49), it is ever more critical to have clear, transparent, and tailored messages regarding the importance of receiving the COVID-19 vaccine and/or booster and addressing vaccine hesitancy. Project *2VIDA!* is a multipronged intervention aimed at addressing this gap in the Southern regions of San Diego which have been heavily impacted by COVID-19. As previously mentioned, Project *2VIDA!* intervention uses evidence-based, CBPR approaches to increase equitable access to COVID-19 information, resources, and pop-up community clinics that provide the vaccine and linkage to healthcare and social services to African American and Latino communities. *2VIDA!* addresses the various limitations of current interventions through the strategic design and implementation grounded in CBPR. Evidence from this intervention will inform efforts to address vaccine hesitancy for COVID-19 and other vaccine preventable infections particularly among communities of color. Vaccination efforts must be multifaceted, responding not only to the culture, history, and values of minoritized communities, but also addressing their concerns by providing reliable information and access to healthcare.

## Ethics statement

The studies involving humans were approved by Human Research Protections Program (HRPP) at the University of California, San Diego (UCSD) and the *Ad hoc* Institutional Review Board (IRB) from San Ysidro Health (SYH). The studies were conducted in accordance with the local legislation and institutional requirements. Written informed consent for participation in this study was provided by the participants’ legal guardians/next of kin.

## Author contributions

BS: Investigation, Methodology, Writing – original draft, Writing – review & editing, Data curation. LS: Conceptualization, Methodology, Writing – review & editing. FM: Conceptualization, Data curation, Formal analysis, Funding acquisition, Investigation, Methodology, Project administration, Resources, Supervision, Validation, Writing – review & editing. GT: Conceptualization, Investigation, Writing – review & editing. DS: Conceptualization, Funding acquisition, Investigation, Methodology, Project administration, Writing – review & editing. JS: Conceptualization, Investigation, Writing – review & editing. SO’B: Project administration, Resources, Supervision, Writing – review & editing. DR: Project administration, Resources, Supervision, Writing – review & editing. CJ-P: Project administration, Resources, Visualization, Writing – review & editing. AS: Conceptualization, Data curation, Formal analysis, Funding acquisition, Investigation, Methodology, Project administration, Resources, Software, Supervision, Validation, Visualization, Writing – original draft, Writing – review & editing.
